# Video-assisted thoracoscopic implantation of a diaphragmatic pacemaker in a child with tetraplegia: indications, technique, and results*

**DOI:** 10.1590/S1806-37132015000100011

**Published:** 2015

**Authors:** Darcy Ribeiro Pinto, Miguel Lia Tedde, Alexandre José Gonçalves Avino, Suzan Lúcia Brancher Brandão, Iuri Zanatta, Rafael Hahn

**Affiliations:** Caxias do Sul University Foundation, Caxias do Sul General Hospital, Department of Thoracic Surgery, Caxias do Sul, Brazil. Department of Thoracic Surgery, Caxias do Sul General Hospital, Caxias do Sul University Foundation, Caxias do Sul, Brazil; University of São Paulo, School of Medicine, Hospital das Clínicas, São Paulo, Brazil. Department of Thoracic Surgery, Heart Institute, University of São Paulo School of Medicine Hospital das Clínicas, São Paulo, Brazil; Caxias do Sul University Foundation, Caxias do Sul General Hospital, Department of Thoracic Surgery, Caxias do Sul, Brazil. Department of Thoracic Surgery, Caxias do Sul General Hospital, Caxias do Sul University Foundation, Caxias do Sul, Brazil; Caxias do Sul University Foundation, Caxias do Sul General Hospital, Department of Thoracic Surgery, Caxias do Sul, Brazil. Department of Thoracic Surgery, Caxias do Sul General Hospital, Caxias do Sul University Foundation, Caxias do Sul, Brazil; Caxias do Sul University Foundation, Caxias do Sul General Hospital, Caxias do Sul, Brazil. Caxias do Sul General Hospital, Caxias do Sul University Foundation, Caxias do Sul, Brazil; Caxias do Sul University Foundation, Caxias do Sul General Hospital, Caxias do Sul, Brazil. Caxias do Sul General Hospital, Caxias do Sul University Foundation, Caxias do Sul, Brazil

**Keywords:** Spinal cord injuries, Respiration, artificial, Pacemaker, artificial, Quadriplegia

## Abstract

We report the case of a child with tetraplegia after cervical trauma, who subsequently underwent diaphragmatic pacemaker implantation. We reviewed the major indications for diaphragmatic pacing and the types of devices employed. We highlight the unequivocal benefit of diaphragmatic pacing in the social and educational reintegration of individuals with tetraplegia.

## Abstract (pt)

Relatamos o caso de uma criança tetraplégica após trauma cervical que foi posteriormente submetida a implante de marca-passo diafragmático. Revisamos as principais indicações da estimulação diafragmática e os tipos de dispositivos empregados, assim como apontamos o inequívoco benefício da reinserção socioeducacional desses indivíduos na sociedade.

## Introduction

The generic term "diaphragmatic pacemaker" (DP) refers to a device that generates electrical impulses delivered to the phrenic nerve in order to produce diaphragm contractions that are aimed at replacing mechanical ventilation in patients with respiratory failure. The key requirement for the use of this treatment approach is that the phrenic nerve is preserved.

Electrical stimulation of the phrenic nerve was described 200 years ago, and, since then, this treatment approach has been investigated under various conditions, such as asphyxia, cholera, polio, and apnea.^(^ 1 ^)^ However, the clinical use of DPs has occurred only in recent decades, after the study by Glenn & Phelps, who implanted a DP into patients with spinal trauma and congenital central hypoventilation syndrome. ^(^ 2 ^,^ 3 ^)^ Since then, there have been many advances in diaphragm pacing, also referred to as electrical^(^ 4 ^)^ or electrophrenic^(^ 5 ^)^ ventilation.

There are two types of devices for stimulation of the phrenic nerve, depending on the site of implantation: directly on the phrenic nerve or directly on the diaphragm. Phrenic nerve pacemakers can be implanted on the nerve via the cervical or thoracic route. The phrenic nerve arises from the C3 and C4 nerve roots, and, more distally, from the C5 nerve root. When implantation is performed via the cervical route, there is the risk of the electrode being positioned on the nerve below its junction to the C5 nerve root. The presence of a tracheostomy can increase the risk of infection.^(^ 6 ^)^ Therefore, the recommended site for implantation is the intrathoracic segment of the phrenic nerve, preferably by means of video-assisted thoracoscopic surgery. 

In addition to implanting electrodes around the phrenic nerve, it is necessary to create a subcutaneous pocket to place a metallic extension of the electrode.

**Figure 1 -:**
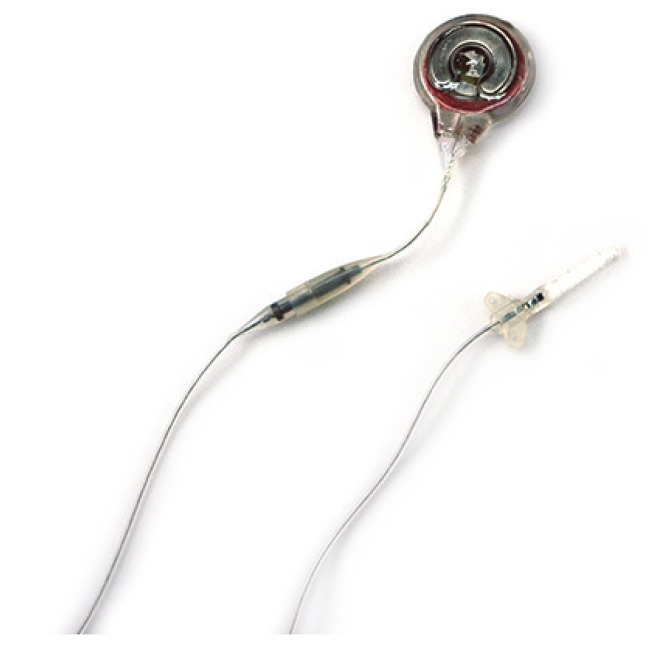
Diaphragmatic pacemaker with the receiver connected to the electrode.

On the international market, there are two models of phrenic pacemakers: Mark IV^(r)^ (Avery Biomedical Devices, Commack, NY, USA), which is monopolar (Figure 1),^(^ 7 ^)^ and Atrostim^(r)^ (Atrotech, Tampere, Finland), which is quadripolar.^(^ 8 ^,^ 9 ^)^

Another device that has been developed more recently is a true DP: NeuRx^(r)^ (Synapse Biomedical, Oberlin, OH, USA).^(^ 10 ^,^ 11 ^)^ This device is laparoscopically implanted directly on the motor point of the diaphragm. Although it has the theoretical advantage of being implanted via a single route, this device has one disadvantage: its electrodes are exteriorized through the skin of the patient.

Such types of pacemakers have been used in three groups of patients. The classic indication is patients with respiratory failure after spinal trauma or with respiratory failure due to central lesions caused by tumors or strokes. Another indication is patients with congenital central hypoventilation syndrome (Ondine syndrome),^(^ 12 ^)^ particularly full-time ventilator-dependent patients, so that they can gain mobility during daytime. Finally, an indication that has yet to be proven is patients with amyotrophic lateral sclerosis, in the hope that DPs can delay the onset of respiratory failure.^(^ 13 ^)^

Considering that the use of electrical ventilation is still limited in Brazil, the objective of the present study was to report a case of DP implantation in a child with spinal trauma, not only to demonstrate the benefits obtained from this treatment option (essentially, the social reintegration of the patient and the technical ease of video-assisted thoracoscopic implantation) but also to demonstrate that DPs can be handled without difficulty even outside the hospital setting.

## Case report

A five-year-old male patient from the city of Caxias do Sul, Brazil, presented with a history of pedestrian-motor vehicle accident (in January of 2010) and an upper cervical spine fracture, at C3-C4. The spinal cord injury was confirmed by axial CT and 3D reconstruction.

The patient underwent alignment and surgical fixation of the corresponding vertebrae. He developed tetraplegia and respiratory failure requiring full-time mechanical ventilation, and underwent tracheostomy, gastrostomy, and cystostomy. In addition to the clinical circumstances, social isolation was crucial to the family's decision to seek help. In social networks, his suffering was shared with other patients experiencing the same condition.

In April of 2013, implantation of a phrenic pacemaker was recommended. The preoperative evaluation included routine hematological tests, arterial blood gas analysis, and chest X-ray. A nerve conduction study of the phrenic nerve was requested in order to assess its functional viability, which could be impaired because of traumatic ischemia, as well as to evaluate phrenic nerve/diaphragm integrity, which is a key requirement for implantation. The nerve conduction study revealed proper nerve conduction, with a good response of the diaphragm to cervical transcutaneous electrical stimulation.

The surgical procedure for implantation of the device was conducted by the team of the Department of Thoracic Surgery of the General Hospital of the University of Caxias do Sul Foundation, in the city of Caxias do Sul, Brazil.

Because of the patient's specific clinical status, the anesthetic and surgical procedure demanded precautions that are unusual in young patients.^(^ 14 ^)^ The patient was given general anesthesia with a tracheal tube inserted through the tracheostomy stoma, with the aid of bronchoscopy to achieve unilateral ventilation during the procedure. With the patient placed in the lateral decubitus position, a 10-mm trocar was inserted in the midaxillary line, sixth intercostal space, for introduction of a fiber optic probe (30º). A 3-cm minithoracotomy was performed in the anterior axillary line, fourth intercostal space, for insertion of the components of the Mark IV^(r)^ system and for phrenic nerve dissection.

**Figure 2 -:**
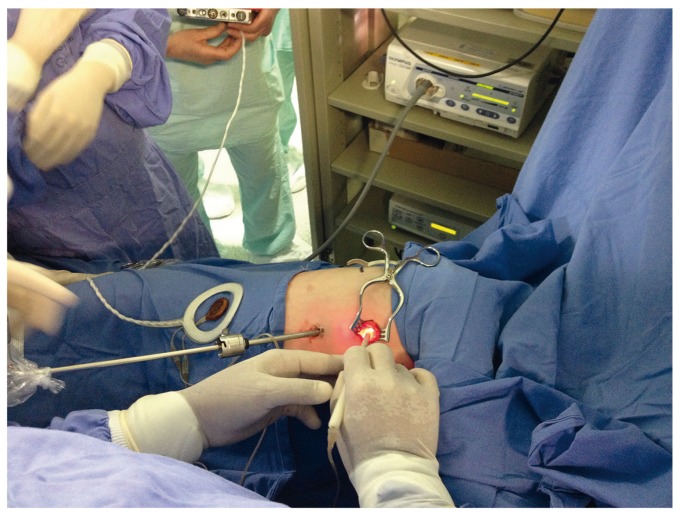
Photograph showing the time of implantation of the diaphragmatic pacemaker intraoperatively.

**Figure 3 -:**
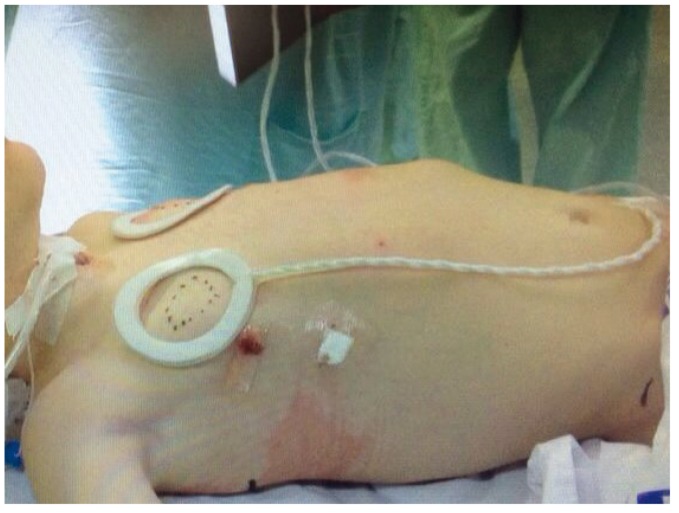
Photograph showing the positioning of the receivers and antenna intraoperatively.

Over the pericardium, the nerve was carefully dissected to prevent rupture or ischemia. The extent of dissection was kept as minimal as possible so that the electrode could "embrace" the nerve without causing excessive traction or compression (Figure 2). At this point, the system was connected, and DP stimulation was tested in vivo to confirm diaphragm contraction (Figure 3).

Through the minithoracotomy, over the pectoralis major muscle, a tunnel incision was made to create a subcutaneous pocket in which to place the device receiver, which was connected to the electrode implanted on the phrenic nerve. The system was tested again, under direct thoracoscopic visualization, using different voltages, to define the voltage that would produce an as-isometric-as-possible contraction bilaterally. Residual pneumothorax was evacuated by performing Valsalva maneuvers, without the need for pleural cavity drainage (Figure 3).

The possibility of performing the procedure by means of video-assisted thoracoscopic surgery reduced morbidity and accelerated postoperative recovery. The procedure was uneventful, and the patient was discharged on postoperative day 3.

Given that the use of mechanical ventilation leads to diaphragm atrophy with conversion of slow type I fibers to fast type II fibers,^(^ 15 ^)^ after three weeks, which is the time required for surgical wound healing and for decrease in the edema at the nerve-electrode interface, the period of DP stimulation to condition the diaphragm was started.

## Discussion

Although mechanical ventilation is the life-sustaining factor in patients with upper spinal trauma, it also has negative characteristics, such as diaphragm atrophy, barotrauma, tracheostomy and tube wounds, speech difficulty, loss of smell, etc. In addition, poorly ventilated posterior lung segments, impaired mucociliary clearance, and accumulation of excess secretion result in a high frequency of respiratory infections, which are the leading cause of death in such patients.^(^ 16 ^)^

Transition from the ventilator to the DP requires a systematic progression, both for the child, so that he/she can adapt as physiologically and comfortably as possible, and the caregiver, who should be able to identify signs of ventilatory effort and fatigue. The child's family was trained to recognize such signs and quickly proved to be able to handle the device.

Closure of the tracheostomy tube and perception of the diaphragm muscle contraction caused discomfort and anxiety, but such symptoms were addressed and were improved at each session of use of the equipment by the family itself.

It is known that, in diaphragm pacing with a DP, there is a relationship between stimulation with high frequencies and neural fatigue/degeneration. Initially, stimulation occurred at low frequencies (< 10 Hz) and at an RR of 12 to 15 breaths/min. Accordingly, time off the ventilator was gradually increased day by day, starting with a 5-minute session every hour.

Tracheostomized mechanically ventilated patients experience loss of smell, which prevents them from differentiating foods and recognizing their taste because of a shift in airflow caused by positive pressure. After initiation of ventilation with the DP, the patient regained normal olfactory function, which also represents a gain in quality of life.^(^ 17 ^)^

Dependence on mechanical ventilation and the consequent reduction in mobility, which leads to social isolation, are factors to be considered, especially because, in general, patients are young, previously healthy individuals who have been abruptly deprived of autonomy. Psychological trauma and hopelessness settle in and are issues that need to be addressed by a specialized team. In this scenario, the resumption of social and educational interaction is the most celebrated result of the procedure.

At this writing, the discharged patient is living in his home, where he receives supportive care from a multidisciplinary team, and has returned to regular classes and started going to the movies and on outings with friends and family.

More than one year after the procedure, the child already remains off mechanical ventilation for more than 10 uninterrupted hours, maintaining a satisfactory respiratory pattern (tidal volume = 277 mL; RR = 18 breath/min; and SpO_2_ = 97%). Full-time pacing can be achieved.^(^ 18 ^)^

Although DPs are a technology that is still expensive in Brazil, they are available in the country, and medical industry and medical research point to an increase in the supply and quality of device options.

In conclusion, the possibility of dispensing with mechanical ventilation, even intermittently, allowing the reintegration of such individuals into society, as well as their inclusion in rehabilitation programs that are more effective and their return to school or work life, is the essence of this therapeutic indication.
